# miRNA Expression in Control and FSHD Fetal Human Muscle Biopsies

**DOI:** 10.1371/journal.pone.0116853

**Published:** 2015-02-18

**Authors:** Débora Morueco Portilho, Marcelo Ribeiro Alves, Gueorgui Kratassiouk, Stéphane Roche, Frédérique Magdinier, Eliane Corrêa de Santana, Anna Polesskaya, Annick Harel-Bellan, Vincent Mouly, Wilson Savino, Gillian Butler-Browne, Julie Dumonceaux

**Affiliations:** 1 Sorbonne Universités, UPMC Univ Paris 06, Center of Research in Myology UM76, Paris, France; 2 INSERM UMRS974, Paris, France; 3 CNRS FRE 3617, F-75013, Paris, France; 4 Institut de Myologie, F-75013, Paris, France; 5 Laboratório de Pesquisas sobre o Timo, Instituto Oswaldo Cruz, Fundação Oswaldo Cruz, Rio de Janeiro, RJ, Brasil; 6 Laboratório de Pesquisa Clínica em DST-AIDS, Instituto de Pesquisa de Clínica Evandro Chagas, Fundação Oswaldo Cruz, Rio de Janeiro, RJ, Brasil; 7 CNRS FRE 3377, Université Paris-Sud CEA Saclay, Gif-sur-Yvette, France; 8 Laboratoire de Génétique Médicale et Génomique Fonctionnelle, INSERM UMRS 910, Aix Marseille Université, Faculté de Médecine de la Timone, Marseille, France; Florida State University, UNITED STATES

## Abstract

**Background:**

Facioscapulohumeral muscular dystrophy (FSHD) is an autosomal-dominant disorder and is one of the most common forms of muscular dystrophy. We have recently shown that some hallmarks of FSHD are already expressed in fetal FSHD biopsies, thus opening a new field of investigation for mechanisms leading to FSHD. As microRNAs (miRNAs) play an important role in myogenesis and muscle disorders, in this study we compared miRNAs expression levels during normal and FSHD muscle development.

**Methods:**

Muscle biopsies were obtained from quadriceps of both healthy control and FSHD1 fetuses with ages ranging from 14 to 33 weeks of development. miRNA expression profiles were analyzed using TaqMan Human MicroRNA Arrays.

**Results:**

During human skeletal muscle development, in control muscle biopsies we observed changes for 4 miRNAs potentially involved in secondary muscle fiber formation and 5 miRNAs potentially involved in fiber maturation. When we compared the miRNA profiles obtained from control and FSHD biopsies, we did not observe any differences in the muscle specific miRNAs. However, we identified 8 miRNAs exclusively expressed in FSHD1 samples (miR-330, miR-331-5p, miR-34a, miR-380-3p, miR-516b, miR-582-5p, miR-517* and miR-625) which could represent new biomarkers for this disease. Their putative targets are mainly involved in muscle development and morphogenesis. Interestingly, these FSHD1 specific miRNAs do not target the genes previously described to be involved in FSHD.

**Conclusions:**

This work provides new candidate mechanisms potentially involved in the onset of FSHD pathology. Whether these FSHD specific miRNAs cause deregulations during fetal development, or protect against the appearance of the FSHD phenotype until the second decade of life still needs to be investigated.

## Background

Facioscapulohumeral muscular dystrophy (FSHD) is an autosomal-dominant neuromuscular disorder and is one of the most common forms of muscular dystrophy with an estimated prevalence of 4–7 cases per 100,000 individuals [1]. FSHD is characterized by onset of weakness in an initially restricted and characteristic distribution, often asymmetric, starting with facial weakness, which is often mild and asymptomatic, and followed sequentially by scapular fixator, humeral, truncal, and lower-extremity weakness [2]. Associated non-muscular symptoms may include sensorineural deafness and retinovasculopathy [3]. The clinical severity is heterogeneous, from asymptomatic individuals to wheelchair-dependent patients [3]. Symptoms usually begin during the first or second decade of life and progress slowly over a normal lifespan [4]. Two loci of the disease have been characterized. The first one concerns 95% of the patients and is located in the sub-telomeric region of chromosome 4 [5–7]: control individuals carry 11 to 100 repeats of a 3.3 kb tandemly repeated sequence named D4Z4, whereas FSHD1 patients carry only 1 to 10 repeats [8]. The second locus is located on chromosome 18 and is mutated in 80% of patients, referred to as FSHD2, in whom mutations in the SMCHD1 gene have been found [9–11]. In both FSHD1 and FSHD2 patients, a chromatin relaxation has been noted, associated with the de-repression of the DUX4 transcription factor DUX4 (reviewed by [12, 13]). However, the molecular mechanisms leading to FSHD are still not fully understood and need to be deciphered. We have recently shown that some molecular hallmarks of FSHD and gene deregulations are already observed in fetal FSHD biopsies [14, 15], demonstrating that even if FSHD is usually described as an adult onset pathology, the disease may involve presymptomatic events such as molecular dysregulations during early myogenesis or differentiation.

Because miRNAs have been described to play important roles in the regulation of myogenesis (reviewed by [16]), we have investigated whether or not miRNA expression is impaired in fetal FSHD biopsies as compared to age-matched fetal control biopsies. The results obtained in the control biopsies allowed us to identify how miRNAs are modulated during normal human muscle development. We have also identified 8 miRNAs which are exclusively expressed in FSHD1 samples with putative targets involved in development and morphogenesis, suggesting that a miRNA deregulation can already be observed during FSHD1 muscle development. Our results suggest that this novel set of miRNAs expressed early in muscle development may be associated with the onset of FSHD1 and could be used as a biomarker for FSHD.

## Methods

### Skeletal muscle biopsies and ethic statement

Muscle biopsies were obtained from quadriceps of FSHD1 and healthy control fetuses from spontaneous or legal therapeutic abortions at 14, 15, 16, 18, 20, 22, 24 and 33 weeks of development (Table 1). The collection of fetal and FSHD1 muscle biopsies was approved by the “Agence Française de la Biomedecine” of the French Ministery of Health in order to have legal access to the biological material in full accordance with the law (research protocol number PFS12–007). Written informed consent from the donors or the next of kin was obtained for the use of the samples in research. All investigations have been conducted according to the principles expressed in the Declaration of Helsinki and the data were analyzed anonymously. The gender of each fetus was determined by PCR for the gene TSPY1 specific for the Y chromosome (TSPY1-Fwd: AATACAGGGCTTCTCATTCCA; TSPY1-Rev: GTTAGATCCTGCGAAGTTGTG).

**Table 1:** Features of the analyzed biopsies.

**Biopsies**	**Genetic Defect**	**Fetus Age**	**Number of D4Z4 Repeats**	**Muscle**	**Sex**
**D14.1**	FSHD	14 w	4.5 D4Z4	Quadriceps	Male
**D14.2**	FSHD	14 w	1.5 D4Z4	Quadriceps	Male
**D15**	FSHD	15 w	4D4Z4	Quadriceps	Male
**D22**	FSHD	22 w	7 D4Z4	Quadriceps	Female
**C14**	None	14 w	-	Quadriceps	Male
**C15**	None	15 w	-	Quadriceps	Male
**C16**	None	16 w	-	Quadriceps	Male
**C18**	None	18 w	-	Quadriceps	Male
**C20**	None	20 w	-	Quadriceps	Male
**C22**	None	22 w	-	Quadriceps	Male
**C26**	None	26 w	-	Quadriceps	Male
**C33**	None	33 w	-	Quadriceps	Male

### FSHD patient reports

The fetal muscle biopsies of the D14.1 fetus were performed by fetopathologists at AP-HP (Assistance Publique—Hôpitaux de Paris). The father was diagnosed with FSHD1 at the age of 23 years and displayed a typical clinical phenotype including facial and scapula fixator muscle weakness. He carries 4 repeated units on chromosome 4qA and presently 2 years later, he still walks but uses a cane. The genetic anomaly was inherited from his father who carried the 4qA D4Z4 contraction. The D14.2 fetus was aborted at 14 weeks of development and carries 1.5 D4Z4 repeats. The D15 fetus was aborted at 15 weeks of development and carries 4 D4Z4 repeats. The D22 fetus was aborted at 22 weeks of development and carries 7 D4Z4 repeats.

### miRNA expression profiling

Total RNA was isolated using Trizol (Invitrogen Life Technologies, Carlsbad, CA, USA) according to the manufacturer’s protocols. The quantity of RNA was determined using a NanoDrop ND-1000 Spectrophotometer (Thermo Scientific, Waltham, MA, EUA) and the quality of the RNA was assessed on a 2100 Bioanalyzer (Agilent Technologies, Santa Clara, CA, USA) using the RNA 6000 Pico LabChip kit (Agilent Technologies, Santa Clara, CA, USA). Reverse transcription (RT) was performed using the TaqMan MicroRNA Reverse Transcription Kit (Applied Biosystems, Foster City, CA, USA) in combination with the Megaplex RT human Primer Pool A and Pool B (Applied Biosystems, Foster City, CA, USA), as described in the manufacturer’s protocol. Global miRNA expression profiling was performed by real-time quantitative PCR on the 7900HT Fast Real Time PCR System (Applied Biosystems, Foster City, CA, USA). We used the TaqMan Human MicroRNA Array panel v3.0, which consists of two microfluidic cards, one for each amplified product from pool A and B. These cards contain primers and TaqMan probes enabling detection of a total of 754 human miRNAs plus endogenous control genes.

### Real-time RT-PCR expression analysis

The fluorescence accumulation data of real-time RT-PCR reactions of each sample were used to fit four parameters sigmoid curves to represent each amplification curve using the library qPCR [17], for the R statistical package version 2.922 (R Development Core Team. 2009). The cycle of quantification was determined for each amplification by the maximum of the second derivative of the fitted sigmoid curve. The efficiency of each amplification reaction was calculated as the ratio between the fluorescence of the cycle of quantification and the fluorescence of the cycle immediately preceding that. The estimated efficiency of each miRNA was obtained by the mean of the efficiencies calculated for each amplification reaction of that precise miRNA. Normalization between the different amplified samples was achieved by the calculation of normalization factors given by the geometric mean of the expression value of all expressed microRNAs in a given sample [18]. The comparisons of means of normalized miRNA expression values between groups were performed by a nonparametric one-way ANOVA with 1,000 unrestricted permutations, followed by post-hoc pair-wise comparisons with Bonferroni adjustment by a nonparametric t-test also with 1,000 permutations [19]. Results were represented in graphs displaying the miRNA expression levels mean ± standard error. Two-tailed levels of significance ≤ 0.01, 0.05 and 0.1 were considered as “highly significant”, “significant” and “suggestive”, respectively.

### Bioinformatics enrichment analysis of miRNA targets

We identified miRNAs potentially involved in FSHD1 onset, based on selective expression or on differential expression between FSHD1 and controls at different stages of fetal muscle development. For these miRNAs, we identified putative target genes based on predictions from five online software programs: Target Scan (http://www.targetscan.org/index.html),miRNAMap (http://mirnamap.mbc.nctu.edu.tw), PicTar (http://pictar.bio.nyu.edu/), Microcosm Target (http://www.ebi.ac.uk/enright-srv/microcosm/htdocs/targets/v5) and miRanda (http://www.microrna.org/microrna/home).

Any target gene was considered a putative target if it was predicted in at least 3 out of the five predicting software’s. In addition, to identify relevant molecular mechanisms potentially associated with the modulation of miRNAs in FSHD samples, we performed a gene set enrichment analysis (GSEA) with putative target genes [20]. A gene set was defined as all putative target genes that share the same ontology based on the Gene Ontology (GO) database [21]. The GSEA method identified biological processes (BP), which were over represented among the list of target genes. The over representation was assessed with a statistical score based on a hypergeometric test with p-values ≤ 0.001.

## Results and Discussion

### myomiR expression signatures during normal fetal skeletal muscle development

To determine whether the miRNA transcriptome was affected in fetal FSHD biopsies, we have profiled the expression of all miRNAs in quadriceps biopsies from 4 fetal FSHD and 8 fetal controls at different stages of development (Table 1). The expression level of a total of 753 mature human miRNAs was assessed.

**Figure 1:**
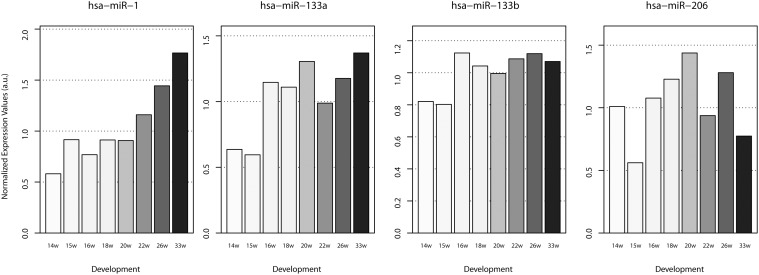
Expression of myomiRs during normal human skeletal muscle development. qPCR for myomiRs were performed on control muscle biopsies at 16–33 weeks of development.

We first analyzed the expression profile of a specific set of miRNAs called myomiRs. The best-studied myomiRs are the miR-1/miR-206 and miR-133a/miR133-b families. All have been previously described to be modulated both during murine embryogenesis and adult muscle regeneration [22, 23] but their expression during human skeletal muscle development have not yet been reported. Interestingly, whereas these 4 miRNAs are dramatically up-regulated during myoblasts differentiation [24], they do not have the same expression profile during human muscle development: miR-1 progressively increased during development, miR-133a/miR-133b were highly expressed from 16 weeks of development until birth and miR-206 was expressed at a similar level at all development stages studied (Fig. 1). In muscle cell cultures, miR-1 and miR-206 promote muscle cell differentiation, whereas miR-133 promotes myoblast proliferation [25]. Since we analyzed whole human biopsies isolated after myotube formation, a high expression of miR-133 was not expected. Our results therefore seem to suggest a different function for miR-133 during human muscle development, as opposed to previous studies in animal models, reinforcing the concept of species-specific miRNA signatures during skeletal muscle development. In contrast, the expression pattern of miR-206 is very similar to what has been reported to occur during mouse embryonic development, where miR-206 was detected at very low levels as early as 9.5 days of development but then increased after secondary muscle fiber formation [26]. During the regulation of skeletal muscle development, miR-1 and miR-206 share similar functions, such as their seed sequences, target genes, and muscle-specific expression patterns. However, during the regulation of myogenesis, miR-1 has more regulatory functions when compared with miR-206 since it has more target genes that are able to influence differentiation [27]. Moreover, it has been demonstrated that MyoD and myogenin bind to regions upstream of miR-1 and miR-206, inducing their expression [28]. The different expression patterns of miR-206 and miR-1 observed during human fetal muscle development suggests a more complex regulation of their expression.

### Expression patterns of myomiRs are not altered in fetal FSHD1 biopsies

**Figure 2:**
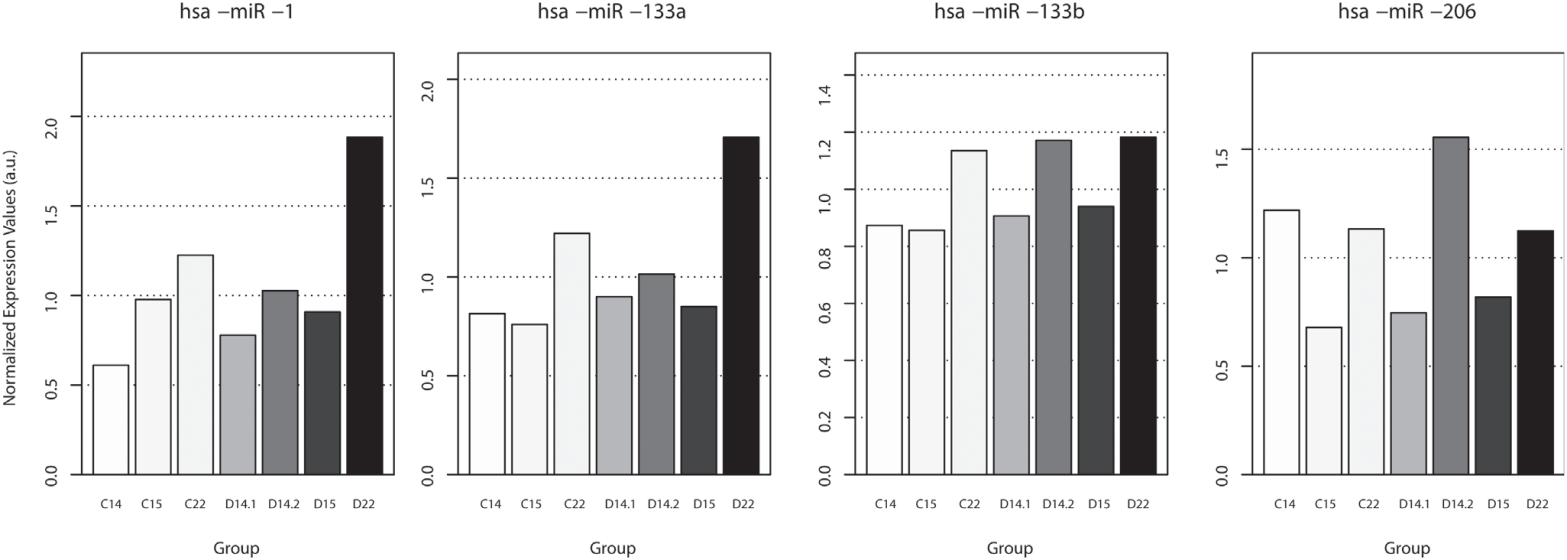
Expression of myomiR in fetal muscle biopsies from FSHD1 are similar to fetal control muscle biopsies. Age-matched pair-wise comparison of myomiRs expression in FSHD1 (D) and healthy control biopsies (C) at 14, 15 and 22 weeks of development.

We next investigated the expression of the myomiRs in FSHD1 samples. Fig. 2 shows that the expression patterns of myomiRs in FSHD1 samples (D14.1, D14.2, D15 and D22) were very similar to those observed in control muscle biopsies (C14, C 15 and C24) during development. This suggests that the D4Z4 contraction does not impact myomiR expression, unlike in Duchenne Muscular Dystrophy (DMD) where miR-1, miR-133a, and miR-206 were highly abundant in the serum of DMD patients but down-regulated in muscle [29, 30]. At first view our results could seem to be in contradiction with previous results showing that miR-133a is up-regulated in FSHD2 myoblasts derived from an adult quadriceps [31]. However, these data were obtained *in vitro* whereas our assays were performed *in* vivo, on FSHD1 fetal muscle biopsies where we have already shown that *DUX4* and DUX4 downstream target genes are expressed [14, 15]. Interestingly, this also strongly suggests that in FSHD1, *DUX4*-dependent transcriptional activation has no impact on myomiRs expression levels.

### Distinctive patterns of miRNAs expression during human muscle development

**Figure 3:**
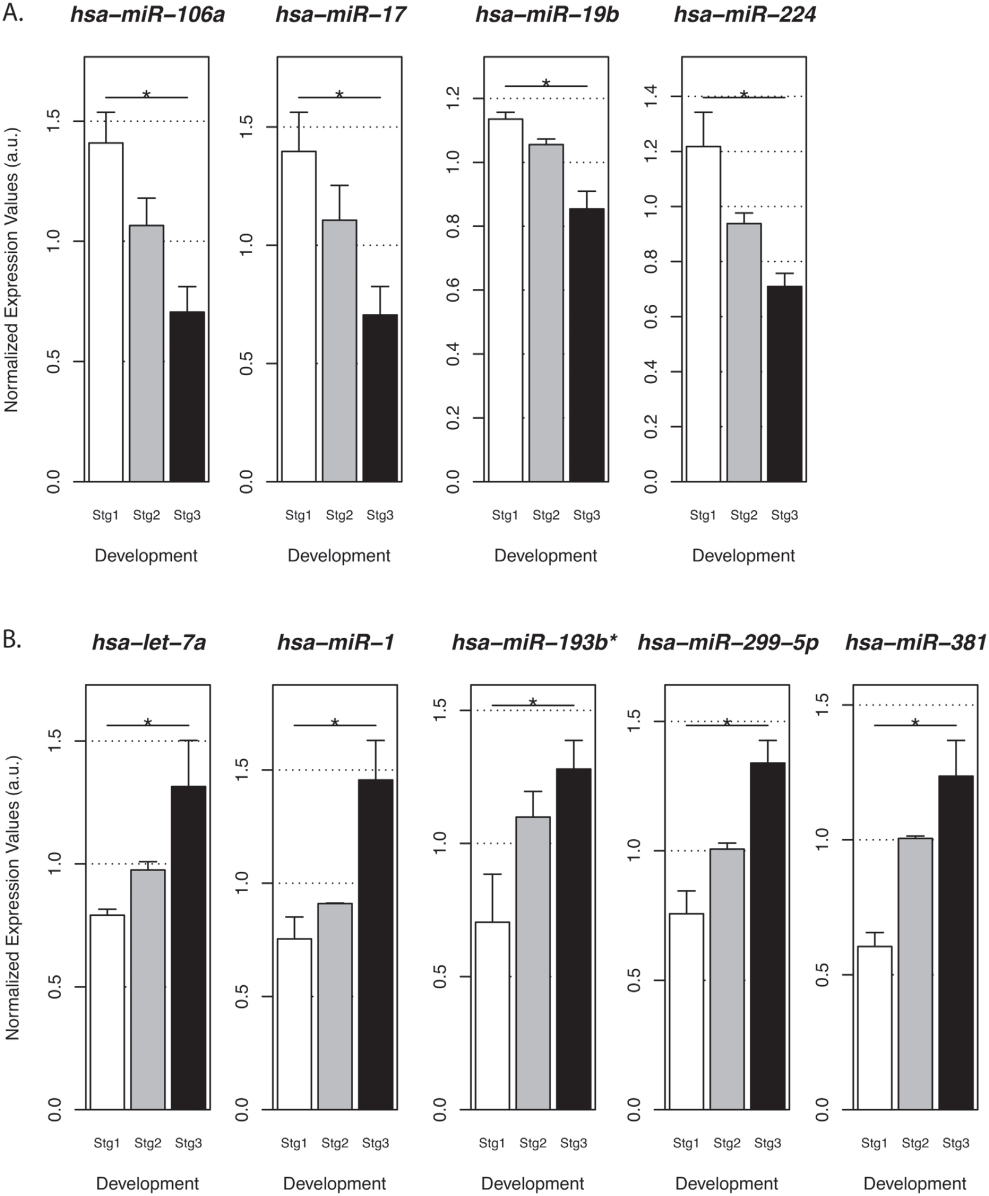
Distinctive patterns of miRNAs expression in human muscular development. Global miRNA expression profiling was performed using control muscle biopsies at 14, 15, 16, 18, 20, 22, 26 and 33 weeks of development. Stg2 (Stage 2, biopsies at 14–16 weeks of development) corresponds to secondary muscle fiber formation; trans (biopsies at 18 and 20 weeks of development) is the transition phase between the secondary myogenesis and the maturation phase; and, Stg3 3 (Stage 3, biopsies at 22–33 weeks of development) corresponds to the maturation phase. A- down-regulated microRNAs during development; B- up-regulated microRNAs during development (* P<0.05; nonparametric one-way ANOVA with 1,000 permutations; compared Stg2 with Stg3).

Skeletal muscle forms in three distinct phases: (i) the first phase corresponds to primary (embryonic) muscle fiber formation (5–10 weeks of development), when the undifferentiated cells become committed to the skeletal muscle program and fuse to form the first generation of muscle fibers; (ii) the second phase corresponds to the secondary (fetal) muscle fiber formation (11–20 weeks of development), when myoblasts align and fuse to form secondary muscle fibers on the surface of pre-existing primary muscle fibers that serve as a scaffold; (iii) finally, during the third phase, the fibers undergo volumetric growth and maturation (21–40 weeks of development) [32–34]. Identification of miRNAs involved in these processes will provide insights into the regulation of muscle development and growth. In order to analyze the variation of miRNAs expression levels during human muscle development, we compared the whole miRNome profile of control biopsies during the different stages of muscle development (Fig. 3A and 3B and S1 Fig.). Among the 754 miRNAs tested, 91 mature miRNAs were not expressed in any of the biopsies suggesting that they were not relevant for skeletal muscle development. Surprisingly, miR-29b, which has been previously described to be involved in muscle differentiation [35, 36] and in porcine skeletal muscle development [35], was not expressed in any sample, suggesting that it may participate to human muscle regeneration events, but is not involved in human muscle development. However, we did find miRNAs previously identified as being differentially expressed during myoblast differentiation, which were modulated during stages 2 and 3 of muscle maturation (including both down-regulated and up-regulated miRNAs). miR-106a, miR-17, miR-19b and miR-224, were highly expressed during secondary muscle fiber formation (Stg2) and then decreased significantly during the subsequent phases of development (Fig. 3A), suggesting that they may be functionally related to the phase of secondary muscle fiber formation. We found a down-regulation of miR-17 during development, which is in accordance with a previous study showing that miR-17 inhibits tissue growth, cell adhesion and proliferation [37]. For miR-19b a recent study showed that overexpression of miR-19a/b was sufficient to induce hypertrophy in neonatal rat cardiomyocytes and revealed that miR-19a/b directly targets the anti-hypertrophic genes atrogin-1 and MuRF-1 (muscle RING-finger protein-1) [38]. Our results also suggest the involvement of miR-146b, miR-149, miR-20a, miR-25 and miR-99b* in secondary muscle fiber formation (S1A Fig.). It has been published that miR-146 targets Numb, which promotes satellite cell differentiation by inhibiting Notch signaling. Inhibition of miR-146 rescued the expression of Numb and facilitated the differentiation of C2C12 [39]. However, our data suggests that miR-146 is important for an efficient secondary myogenesis during human muscle development. The expression level of miR-20a decreased during muscle maturation. Interestingly, Brock *et al*. have recently shown that this miRNA is a regulator of *BMPR2* expression, a member of the BMP pathway known to act as an inhibitor of myogenic differentiation [40]. These findings support the idea that miR-20a may target BMP receptor transcripts, thus inhibiting the BMP pathway, ultimately favoring the formation of secondary fibers.

In contrast, miR-1, miR-299–5p, miR-381, miR-193b* and let-7a seem to be more associated with the third phase of muscle development, the volumetric growth and maturation of the muscle fibers (Stg3, Fig. 3B). Interestingly, similar to what has been found in porcine muscle development, miR-299–5p was up-regulated during human muscle development. Recently, let-7a was identified to be expressed at a moderate to high level in many fetal organs, like brain, liver, kidney and heart [41]. We showed that its expression is also high during skeletal muscle maturation, which is in accordance with the data showing that the let-7 family reduces cell proliferation [42] and could be involved in the metabolic maturation of the muscle fibers. Our results also suggest the involvement of miR-127, miR-27b, miR-30b, miR-655, miR-95 and miR-495 in skeletal muscle development (S1B Fig.). For example, miR-27b was described to down-regulate PAX3 protein levels ensuring robust entry into the myogenic differentiation program in mice [43]. Our data also showed an increase of this microRNA during skeletal muscle development, suggesting its involvement in skeletal muscle growth. miR-655, which seems to be increased during muscle maturation, was not described as being modulated in pig muscle development [35]. Recently, miR-30b has been reported to act as a hypertrophic suppressor by inhibition of Ca/calmodulin-dependent protein kinase II expression (CaMKII), a hypertrophic signaling marker [44]. However, our data demonstrated that miR-30b might be important to volumetric muscle growth and muscle maturation.

We did not observe any statistically significant variation in the expression levels of miR-329, miR-422a, miR-493 during development whereas these miRNAs have been described to be modulated during porcine muscle development [35]. Similarly, we did not find any difference in miR-214 expression during human muscle development, although it has been reported to promote the slow muscle phenotype in zebrafish [45] suggesting that it could have been up-regulated during secondary muscle fiber formation. We also detected some differentially expressed miRNAs during human skeletal muscle development, such as miR-224, and miR-381, which have not yet been associated with muscle formation.

Our results also identify a number of differentially expressed miRNAs that could represent new regulatory factors in muscle growth and development. Overall, these findings highlight the concept of a species-specific miRNA signature during muscle development. The fact that the miRNAs are differentially expressed during the different stages of muscle development confirms the complexity of this fine tuning to coordinate the formation and maturation of the human skeletal muscle.

### MicroRNAs differentially expressed between FSHD1 and healthy fetal muscle

**Figure 4:**
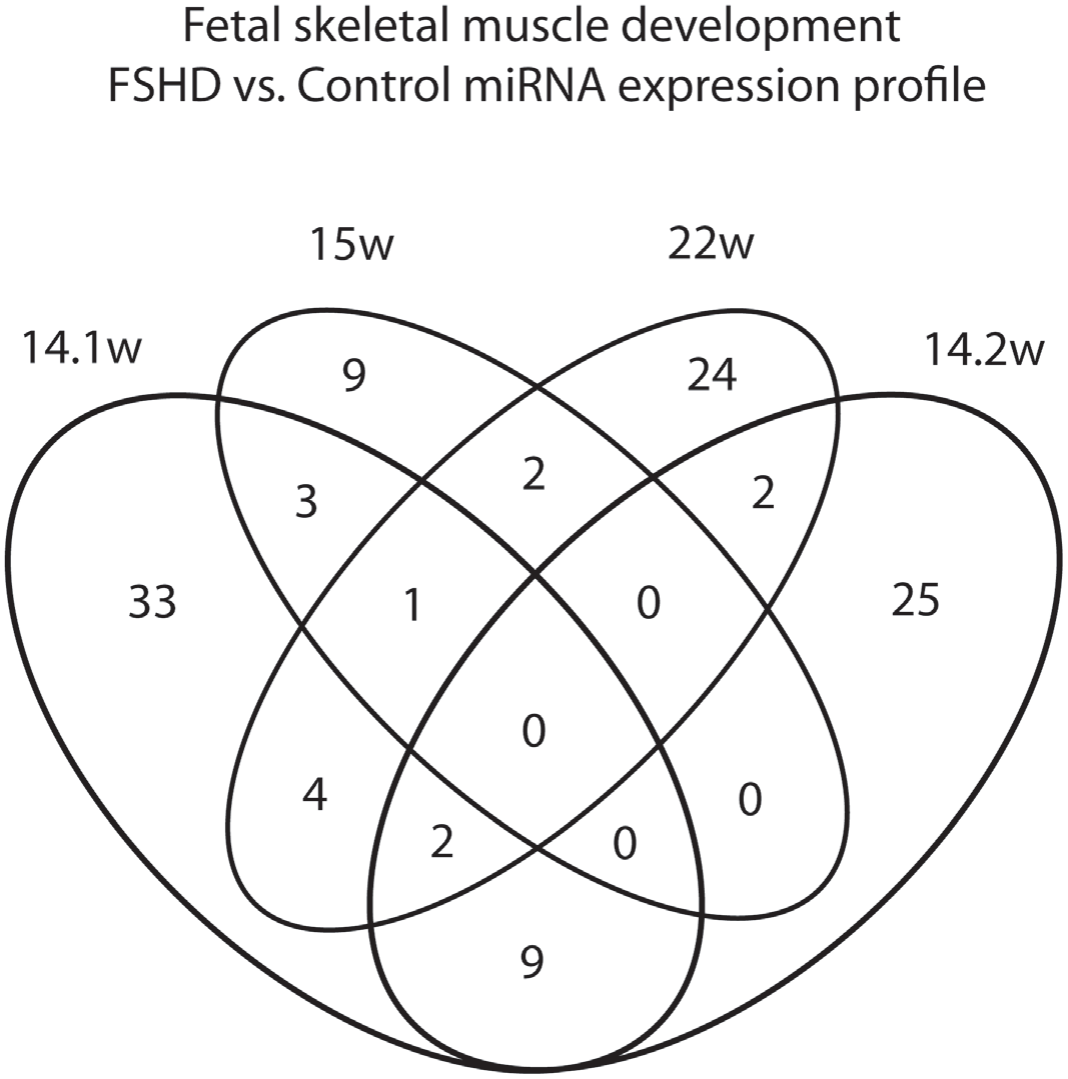
Venn-diagram showing the number of miRNAs modulated in FSHD1 fetuses at different stages of development. 52 miRNAs were differentially expressed in the FSHD1 fetus at 14 weeks of development carrying 4.5 D4Z4 repeat units (FSHD 14.1), while 38 microRNAs were modulated in the FSHD1 fetus at 14 weeks of development with 1.5 D4Z4 repeat units (FSHD 14.2). These two fetuses at the same stage share 11 modulated miRNAs. 15 miRNAs were modulated in the FSHD1 fetus at 15 weeks of development, while at 22 weeks of development, we observed 35 differentially expressed miRNAs in FSHD1.

Considering the difficulty in obtaining FSHD1 samples, we carried out an age-matched pair-wise comparison with corresponding normal samples (S1 Table). These data are summarized in Fig. 4, Table 2 and S2 Fig. Four FSHD1 muscle biopsies were analyzed (Table 1).

**Table 2:** Summary of differentially expressed miRNAs.

**Comparison Groups**	**Direction**	**>=1.5fold**	**>=2fold**	**>=3fold**	**>=5fold**	**>=10fold**	**MedianFC**
14.1(FSHDxContr)	Up	35	10	0	0	0	1.758541
14.1(FSHDxContr)	Down	17	10	7	6	4	0.436243
14.1(FSHDxContr)	All	52	20	7	6	4	1.615112
14.2(FSHDxContr)	Up	19	10	5	2	2	2.160025
14.2(FSHDxContr)	Down	19	6	4	3	3	0.590754
14.2(FSHDxContr)	All	38	16	9	5	5	1.083219
15(FSHDxContr)	Up	7	1	1	0	0	1.652615
15(FSHDxContr)	Down	8	3	2	1	0	0.526006
15(FSHDxContr)	All	15	4	3	1	0	0.636391
22(FSHDxContr)	Up	18	7	3	3	1	1.801825
22(FSHDxContr)	Down	17	4	0	0	0	0.613786
22(FSHDxContr)	All	35	11	3	3	1	1.508734

Contr: age-matched control biopsies

The case report evidences for FSHD 14.1 (4.5 D4Z4 repeat units) showed that 52 miRNAs were differentially expressed: 35 were up- and 17 were down-regulated compared to the control biopsies whereas in FSHD14.2 (1.5 D4Z4 repeat units) 38 microRNAs were modulated: 19 were up- and 19 were down-regulated compared to the control biopsies. These two FSHD1 fetal muscle biopsies share 11 miRNAs with similar modulations (Fig. 4), miR-1225–3p, miR-19b-1*, miR-208b, miR-22, miR-372, miR-383, miR-767–3p, miR-802, miR-872, miR-875–5p, and miR-892b. The roles of these miRNAs in skeletal muscle physiology are unknown except for miR-208b, which has been described to play a role in muscle identity by activating slow and repressing fast myofiber gene program [46]. This suggests that FSHD1 fetal biopsies, where miR-208b was down-regulated, could develop defects in muscle fiber specification. This is in agreement with a recent article showing that sarcomeric dysfunction contributes to muscle weakness in facioscapulohumeral muscular dystrophy [47]. miR-628–5p was modulated in FSHD1 at all 3 different fetal ages. However, the function of this miRNA in muscle development and FSHD remains unknown and needs to be deciphered.

15 miRNAs were modulated in the FSHD1 fetal muscle at 15 weeks of development (FSHD 15), among them, 7 were up-regulated and 8 were down-regulated. Despite the fact that this fetus has a similar number of D4Z4 contractions as the FSHD 14.1, they share only four modulated miRNAs (Fig. 4); miR-195, miR-451, miR-1260 and miR-628–5p. Finally, at 22 weeks of development, 35 differentially expressed miRNAs were identified in FSHD muscle, with 18 up-regulated and 17 down-regulated. Although differences in miRNA expression were observed between FSHD and healthy samples at different stages of development, no common miRNA was consistently modulated in FSHD during the whole fetal period analyzed (Fig. 4). Finally, we did not observed any consistent modulation of miRNAs recently described as being modulated in FSHD myoblasts [48, 49] probably because our experiments were performed on biopsies and not on cultured cells.

### MicroRNAs exclusively expressed in FSHD fetal muscle

We did not observed any miRNAs only detected in control samples but we identified 8 miRNAs which were only expressed in FSHD samples as compared to age-matched controls: miR-330, miR-331–5p, miR-34a, miR-380–3p, miR-516b, miR-582–5p, miR-517* and miR-625. They were exclusively expressed in at least three FSHD muscles (Table 3) and none of them was expressed in control biopsies. Moreover, they are not present on chromosome 4q35 where the D4Z4 repeats are located. It is interesting to note that miR-34a has been previously reported to be up-regulated in adult biopsies isolated from FSHD1, DMD, limb girdle muscular dystrophy 2a and 2b, Miyoshi myopathy and type 2 myotonic dystrophy patients [50–52], suggesting its involvement in a deregulated pathway common to many muscular diseases. Eisenberg *et al*. also showed that miR-517* was up-regulated only in adult FSHD1 biopsies and not in any of the 9 other neuromuscular diseases studied by these authors, suggesting that this microRNA might be a potent biomarker for FSHD [51]. Recently, miR-331 has been reported to be up-regulated in FSHD primary myoblasts [53]. The role of these four miRNAs in muscle still remains to be determined. The 4 other miRNAs have not been previously described in FSHD1 and may be specific to the deregulation which occurs during human FSHD1 muscle development.

**Table 3:** microRNAs exclusively expressed in FSHD biopsies.

**Only expressed in FSHD**	**Localization**
hsa-miR-330	Chr 19
hsa-miR-331–5p	Chr 12
hsa-miR-34a	Chr 1
hsa-miR-380–3p	Chr 14
hsa-miR-516b	Chr 19
hsa-miR-582–5p	Chr 5
hsa-miR-517*	Chr 19
hsa-miR-625	Chr 14

The relation between the mis-regulation of these 8 miRNAs only expressed in the FSHD biopsies and DUX4 was also investigated. We did not chose to overexpress DUX4 in control cells and to investigate the expression of these miRNAs in these conditions because (i) overexpression is an artificial system which may not correctly mimic what really happens, (ii), several articles have already compared control and FSHD myoblasts or myotubes (when DUX4 is expressed) and did not observed a modification of the expression of these miRNAs (except for miR-331 in one of them) [48, 49, 53]. Finally, we also have made an in silico analysis to look for miRNA putatively regulated by DUX4. We have downloaded the TransmiR database [39] v.1.2 (last updated in 2013–1–30), and queried for DUX4 (entrezid 22947). Apparently, neither DUX4 nor any gene regulated by it, as described in Geng et al. [54], is responsible for regulating any of these miRNA. We conclude that the expression of these 8 miRNAs is not linked to DUX4 expression.

One possible explanation is the involvement of another gene in FSHD onset which may mis-regulate some miRNAs in fetal samples. We have recently shown that FAT1 is lower expressed in fetal FSHD muscle biopsies compared to age matched control fetal biopsies [55]. We have also shown that a transgenic mouse down-regulating the planar polarity gene Fat1 exhibits most of the phenotype observed in FSHD patients. We have shown in this mouse an early function of Fat1 in patterning myoblast migration and muscle shape in subsets of muscles in the scapulohumeral region. This lower expression of FAT1 in fetal samples could be linked to a mis-regulation of several miRNAs.

### Predicted targets of exclusive FSHD1 microRNAs

**Figure 5:**
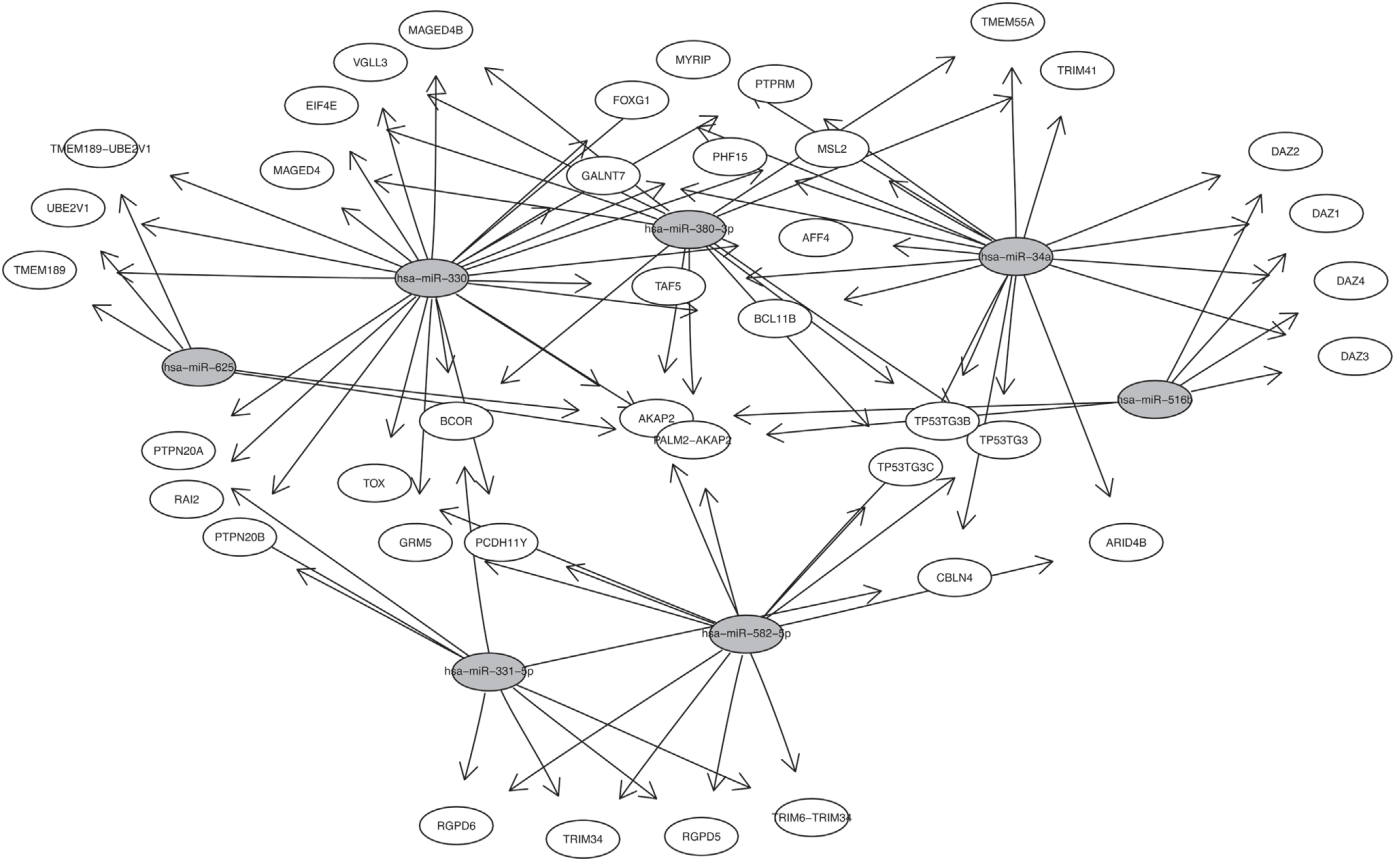
Predicted common targets of miRNAs expressed exclusively in FSHD1. Five different algorithms were used to identify the predicted targets for miRs exclusively expressed in FSHD1 biopsies, and only the target-genes predicted in at least 3 of them were considered as probable target. In gray, miRNAs exclusively expressed in FSHD1 fetal muscle biopsies and in white, target genes of these FSHD1 miRNAs.

Available prediction algorithms usually predict hundreds of potential target genes for a single miRNA and often generate false-positive candidates. In order to reduce such a high number of theoretical targets, and to make a more reliable prediction, we applied five different algorithms, and considered as potential targets only the genes that were predicted by at least 3 of these algorithms. We first checked if the FSHD1 exclusive miRNAs can target some genes previously described to be involved in FSHD pathology including *DUX4*, *FRG1*, *FRG2*, *ANT1*, *CRYM* and *FAT1* [56–58] but none of them appeared in our list. We found that the miRNAs exclusively expressed in FSHD1 fetal muscle biopsies were predicted to target 632 genes, involved in a variety of functions (S2 Table). Among these 632 genes, 40 could be modulated by more than one miRNA (Fig. 5). For example, miR-516b, miR-582–5p and miR-625 target *AKAP2*, which was identified in skeletal muscle, brain and testis, to be involved in the cAMP signaling pathway [59]. *RAI2*, which is a target of miR-330 and miR-331–5p, is described to be involved in development and is up-regulated in skeletal muscle side population cells. Finally, the specific FSHD microRNAs are predicted to down-regulate *TRIM2*, *TRIM3*, *TRIM6*, *TRIM34* and *TRIM41*. The TRIM family has been implicated in cell differentiation, apoptosis, transcriptional regulation and signaling pathways [60] and the FSHD miRNAs could thus interfere with these biological processes in FSHD fetal muscle.

It should also be considered that this specific set of miRNAs may be expressed to protect muscle. For instance, DUX4 has been described to promote TP53 expression [61] and to induce apoptosis via the p53 pathway [62]. However, our data suggests that in FSHD fetal muscles that express DUX4, TP53 could be down-regulated by the expression of miR-34a, miR-380–3p and miR-582–5p. Another example is *ID2* gene. ID2 has been described to be induced by DUX4 [54], but is also a predicted target of miR-330, which is specifically expressed in FSHD samples These events suggest an alternative mechanism where some of these miRNAs could play a protective role during fetal development and delay the appearance of the FSHD phenotype until development and post-natal growth is finished.

**Figure 6:**
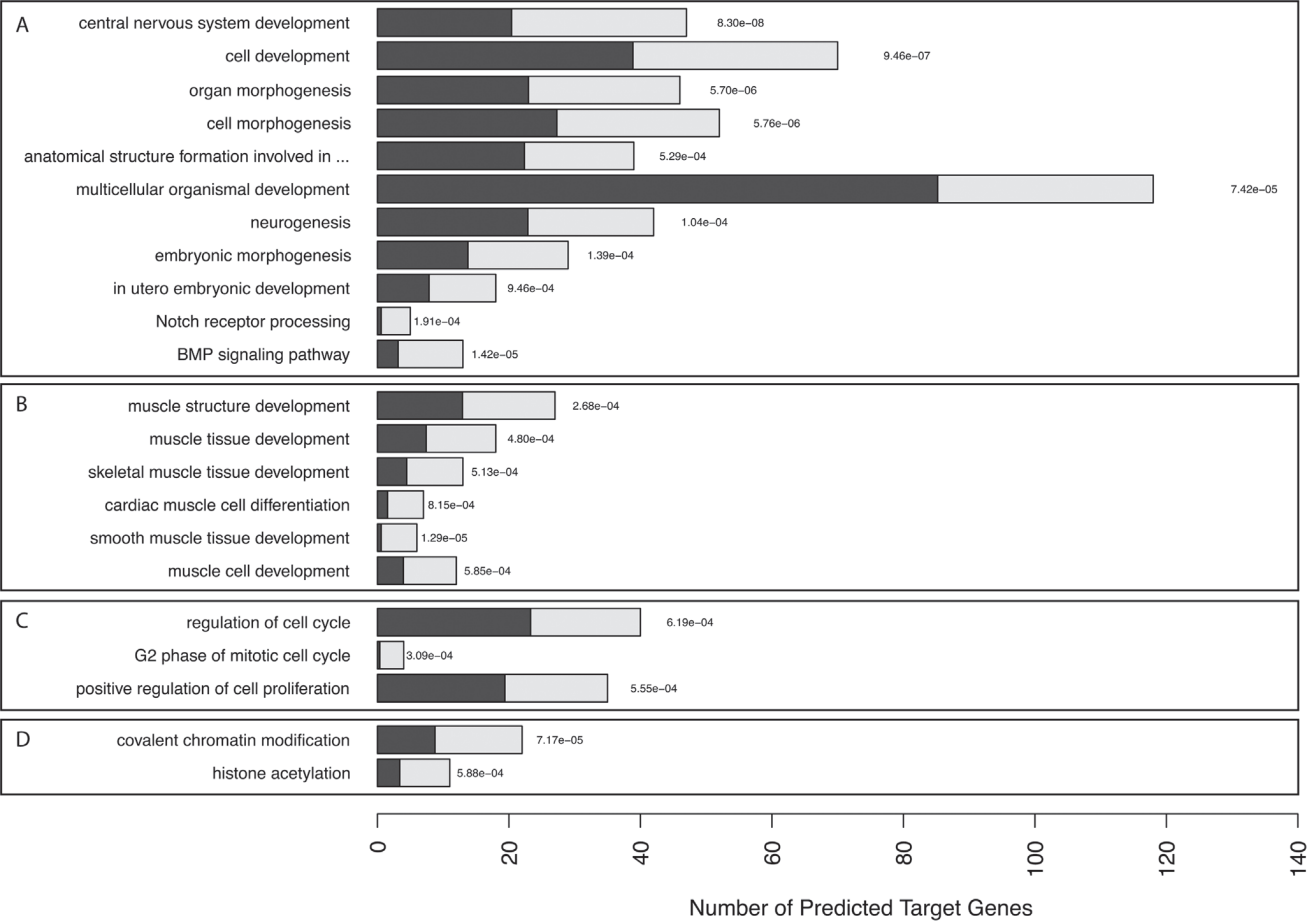
Overrepresented gene ontology-related biological processes in FSHD muscle. Graphical representation of the selected enriched biological processes (BP) from the list of putative target-genes based on Gene Ontology annotation. Dark bars: expected count of putative target-genes in each BP; light bars: observed BP enrichment; and, values alongside the bars. The overrepresentation was assessed with a statistical score based on a hypergeometric tests with p-values ≤ 0.001.

To better analyze the roles that these miRNAs could play in the regulatory networks in FSHD, we identified which biological processes were enriched in our list of predicted down-regulated target-genes (Fig. 6 and S3 Table). A stringent approach to considering over represented Gene Ontology terms with adjusted *p*-values ≤ 0.001 indicated that 97 biological processes were significantly overrepresented in FSHD biopsies. Among them, are processes related to development and morphogenesis, suggesting that affected FSHD muscles may present subtle developmental deregulations due to the expression of the FSHD-specific set of miRNAs. Although no major developmental defects have been described in FSHD, one should keep in mind that these potential defects might be minor and difficult to observe. As seen in Fig. 6, we identified biological processes related to muscle: muscle cell development (GO: 0055001), muscle tissue development (GO: 0060537), muscle structure development (GO: 0061061), skeletal muscle tissue development (GO: 0007519), cardiac muscle cell differentiation (GO: 0055007), and smooth muscle tissue development (GO: 0048745).

Epigenetic regulation is involved in FSHD and it has been shown that FSHD chromosomes have a 30–40% reduction in DNA methylation at specific sites in D4Z4 [63, 64]. We also identified biological processes involved in chromatin modification (GO: 0016569) and histone acetylation (GO: 0016573). Recently it has been described that the biological processes most affected in FSHD patients are involved in histone acetyltransferase [50] and the gene *SUV39H1*, a histone methyl-transferase involved in D4Z4 H3K9me3, was down-regulated in FSHD cells [31]. This histone perturbation may affect chromatin structure and contribute to the epigenetic defect of the D4Z4 array specifically in FSHD patients. Finally, we identified some biological processes involved in cell cycle regulation (GO: 0051726, GO: 0000085, GO: 0008284). Several studies have shown a significant gene deregulation linked to cell cycle control in FSHD cells, essentially affecting G1/S and G2/M transitions [31, 65].

## Conclusions

In this study, we demonstrated that myomiRs expression is not impaired in fetal FSHD1 compared to age-matched control biopsies. However, some non-myomiRs exhibit a mis-regulation in fetal FSHD1 biopsies. In particular, 8 miRNAs were only expressed in FSHD1 samples with putative targets involved mainly in development and morphogenesis. We now need to be determined whether these FSHD specific miRNAs cause deregulations during fetal development, or on the contrary protect against the appearance of the FSHD phenotype until the second decade of life. This study provides new candidate mechanisms potentially involved in the onset of FSHD pathology.

## Supporting Information

S1 Fig Suggestive miRNAs involved in human muscular development. A- down-regulated microRNAs during development; B- up-regulated microRNAs during development (* P<0.1; nonparametric one-way ANOVA with 1,000 permutations; compared Stg2 with Stg3)
(TIF)

S2 Fig Modulated microRNAs in FSHD muscle biopsies at different weeks of development. Fold Change (FC) = 1.5
(TIF)

S1 Table Normalized microRNAs expression data. (XLSX)

S2 Table List of the 632 predicted targets for the exclusive FSHD microRNAs (xls). (XLSX)

S3 Table Biological processes significantly enriched in the set of predicted targets of FSHD exclusive microRNAs (xls). (XLSX)
