# Supplementary figures and images for: miRNA Expression in Control and FSHD Fetal Human Muscle Biopsies
Source: PLoS One. 2015 Feb 18;10(2):e0116853. doi: 10.1371/journal.pone.0116853 (PMC4333765; doi:10.1371/journal.pone.0116853)

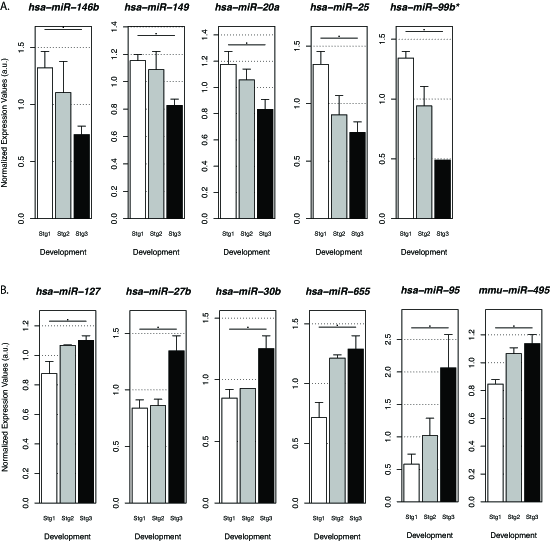

Supplement: S1 Fig — A- down-regulated microRNAs during development; B- up-regulated microRNAs during development (* P<0.1; nonparametric one-way ANOVA with 1,000 permutations; compared Stg2 with Stg3) (TIF) [file pone.0116853.s001.tif]

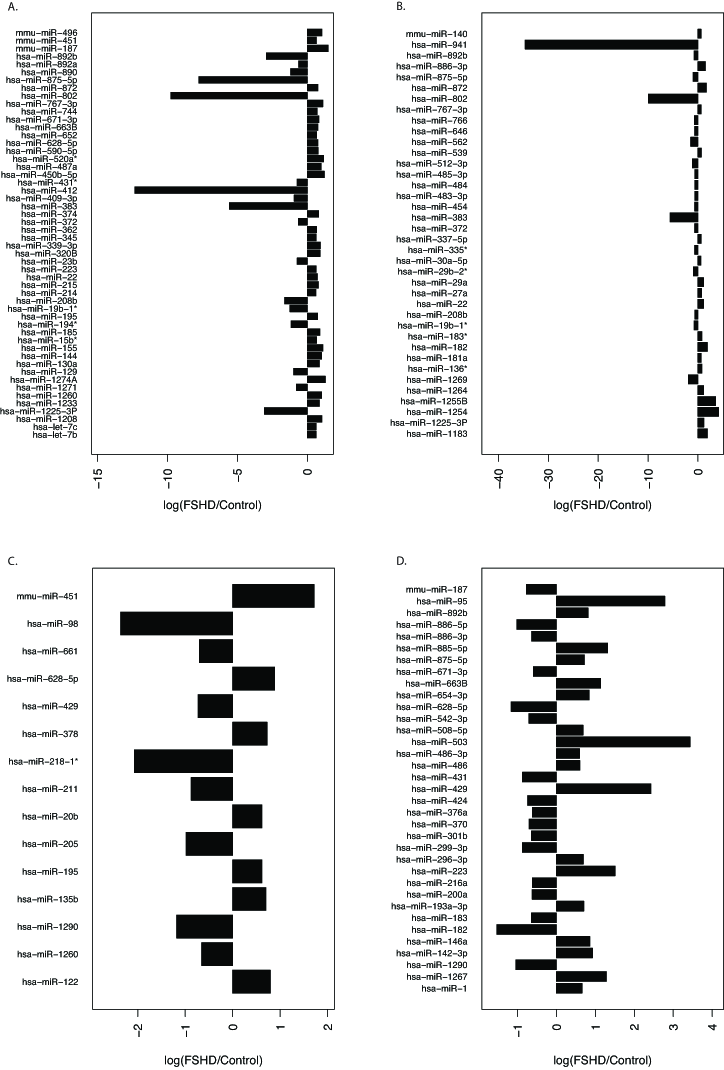

Supplement: S2 Fig — Fold Change (FC) = 1.5 (TIF) [file pone.0116853.s002.tif]
